# Substitution of ifosfamide for cyclophosphamide in the TI-CE regimen: pharmacology and dose justification in a patient with refractory metastatic germ-cell tumor post-nephrectomy—A case report

**DOI:** 10.1007/s00280-025-04818-0

**Published:** 2025-10-22

**Authors:** Lotte M. G. Hulskotte, Loek A. W. de Jong, Alwin D. R. Huitema, Joost Sijm, Ingrid Desar, Minke Smits, Nielka P. van Erp

**Affiliations:** 1 Department of Pharmacy, Pharmacology and Toxicology, Radboud University Medical Center, Nijmegen, The Netherlands; 2 Department of Pharmacy and Pharmacology, The Netherlands Cancer Institute, Amsterdam, The Netherlands; 3 Department of Pharmacology, Princess Máxima Center for Pedriatric Oncology, Utrecht, The Netherlands; 4 Department of Clinical Pharmacy, University Medical Center Utrecht, Utrecht University, Utrecht, The Netherlands; 5 Department of Medical Oncology, Radboud University Medical Center, Nijmegen, The Netherlands

**Keywords:** Germ-cell tumor, ASCT, Nephrectomy, TI-CE regimen, Case report

## Abstract

**Background:**

High-dose chemotherapy (HDCT) combined with autologous stem cell transplantation (ASCT) rescue is an effective treatment option for relapsed or refractory germ-cell tumors. The TI-CE regimen, consisting of paclitaxel and ifosfamide for stem cell mobilization followed by high dose carboplatin and etoposide with ASCT rescue, is frequently used in the treatment of refractory disease. This regimen is challenging in patients who have undergone unilateral nephrectomy, since potential nephrotoxicity of ifosfamide poses a serious risk for permanent damage of the preserved kidney. Currently, the literature lacks data on the substitution of ifosfamide in the TI-CE regimen for an alternative chemotherapeutic agent with equivalent potency while being less nephrotoxic.

**Case presentation:**

We present a case of a patient with refractory progressive metastatic germ-cell tumor who underwent nephrectomy and was successfully treated with a modified chemo-mobilization strategy. In the TI-CE protocol, ifosfamide was replaced for cyclophosphamide (TC-CE), resulting in a sufficient stem cell harvest through a single apheresis session. Post chemo-mobilization, LDH and hCG + hCGβ levels were normalized. Renal function remained stable throughout the course of treatment. Two months after HDCT, the patient showed a complete metabolic response, with no detectable tumor remnants. Currently, one year post-therapy, there are no signs of disease recurrence.

**Conclusion:**

An effective and potentially less nephrotoxic chemo-mobilization regimen, using paclitaxel and cyclophosphamide (TC-CE), was administered to a patient with refractory metastatic germ-cell tumor who underwent HDCT followed by ASCT rescue after unilateral nephrectomy. Cyclophosphamide demonstrated to be a viable substitute for ifosfamide within the TI-CE regimen.

## Introduction

Seminoma, a type of germ cell tumor (GCT) predominantly affecting males aged 18–40 years, is a relatively rare but mostly curable malignancy [1]. The prognosis is normally good, with a 5-year survival rate of approximately 95% [1]. In patients with stage IIC good-risk testicular seminoma, orchidectomy is followed by first-line chemotherapy. This treatment consists of three cycles of cisplatin, etoposide and bleomycin (BEP), or four cycles of cisplatin and etoposide (EP) in cases where bleomycin is contraindicated. Upon disease relapse following first-line chemotherapy, the standard salvage treatment involves four cycles of cisplatin, ifosfamide, and paclitaxel (TIP), associated with a 5-year overall survival (OS) rate of 72% [2]. In cases of poor prognosis with conventional salvage therapy—such as in patients with germ cell tumors exhibiting progressive disease and an incomplete response following first-line chemotherapy—a more advanced therapeutic strategy may be considered [3].

HDCT followed by ASCT rescue has emerged as a pivotal treatment modality for patients with relapsed or refractory GCT, including seminoma [3, 4]. This strategy leverages the cytotoxic potential of high-dose chemotherapeutic agents to eradicate malignant cells, followed by autologous hematopoietic stem cell transplantation to restore bone marrow function [4]. Patients with GCT who exhibit progressive disease following cisplatin-based chemotherapy and have poor prognostic factors, such as an incomplete response to first-line chemotherapy, demonstrate a 5-year OS rate of 52% [3]. The TI-CE regimen, frequently used as HDCT regimen in patients with recurrent germ cell tumors, involves two chemo-mobilization cycles with a two week interval consisting of 200 mg/m² paclitaxel on day 1 and 2000 mg/m²/day ifosfamide on days 1–3 and leukapheresis on day 11–14. Hereafter, the regimen extends to three cycles of HDCT with carboplatin AUC 8 and etoposide 400 mg/m^2^, administered every three weeks on 3 consecutive days starting four days prior to ASCT rescue [3, 4].

The primary objective of utilizing HDCT and ASCT rescue is to attain long-lasting remission and, ideally, eradicate the tumor. Unilateral nephrectomy complicates the clinical management of testicular cancer when using the TI-CE regimen, due to the increased risk of nephrotoxicity associated with ifosfamide [5, 6]. Current literature lacks comprehensive data on considerations and the efficacy of substituting ifosfamide with cyclophosphamide for chemo-mobilization within the TI-CE protocol (TC-CE). This case report outlines the alternative TC-CE regimen in a patient with refractory testicular seminoma post-unilateral nephrectomy, resulting in an adequate stem cell harvest after a single apheresis session. The data presented can serve as a valuable reference point for similar cases.

## Case presentation

A 42-year-old male patient was diagnosed with stage IIC good risk testicular seminoma with hydronephrosis due to retroperitoneal tumor obstruction of the right distal ureter. He underwent a right-sided orchiectomy, and a nephrostomy catheter was placed. Subsequently, he received four cycles of cisplatin 20 mg/m^2^ and etoposide 100 mg/m^2^ from days 1 to 5 every three weeks, supplemented with Granulocyte-Colony Stimulating Factor (G-CSF). Two months after therapy, CT imaging showed a reduction in tumor burden within the right para-aortic region, with persistent residual involvement of the aortocaval lymph nodes. Subsequent PET-CT imaging performed three months later revealed increased metabolic activity and progression of lymphadenopathic disease, specifically within a confluent nodal mass adjacent to the distal ureter. Nine months after the initial diagnosis, the patient underwent retroperitoneal lymph node dissection (RPLND), which yielded clear surgical margins. Histopathological examination revealed that out of seven lymph nodes, two contained seminoma cells, four were completely necrotic, and one was free of tumor involvement. Additionally, unilateral nephrectomy was performed due to a nonfunctioning kidney with a renal clearance of less than 1 mL/min which was susceptible for infections. The residual urinary output was 117 mL/min, indicating adequate renal clearance of the remaining kidney. Two months postoperatively, tumor markers increased rapidly, with human chorionic gonadotropin (hCG + hCGβ) levels rising from less than 2.6 E/L to 18 E/L and lactate dehydrogenase (LDH) levels increasing from 208 U/L to 1495 U/L. In addition, CT-imaging revealed metastatic involvement in the lymph nodes, peritoneal cavity and omental tissue. Due to the rapid progression of disease and incomplete response to first-line platinum-based chemotherapy, the patient was diagnosed with refractory extragonadal seminoma. Initial salvage therapy with HDCT was deemed a potentially more effective curative approach compared to standard second line treatment with TIP (cisplatin, ifosfamide and paclitaxel), which may be followed by HDCT as third-line option [7, 8]. Hence, HDCT supported by ASCT rescue was selected as the second line therapy.

The conventional TI-CE regimen was considered unfeasible, since unilateral nephrectomy and previous exposure to cisplatin are two risk factors for the development of ifosfamide-induced nephrotoxicity [5]. Therefore, the chemo-mobilization regime was adapted to two cycles of paclitaxel 200 mg/m^2^ on day 1 and cyclophosphamide 1300 mg/m^2^ and mercaptoethanesulfonic acid (MESNA) 1950 mg/m^2^ on day 1, 2 and 3 every two weeks, corresponding to a total percentage of 150% of the cyclofosfamide dose. Of the total dose of MESNA, 300 mg/m^2^ was administered prior to the cyclophosphamide administration. During cyclophosphamide administration, 650 mg/m^2^ MESNA was administered, followed by two subsequent doses of 500 mg/m^2^, each with an infusion duration of 8 h. Additionally, G-CSF was administered twice a day in doses of 480 mcg and 300 mcg from day 4–10 to promote the proliferation of hematopoietic stem cells. Ten days after initiating the first chemo-mobilization cycle, the patient developed febrile neutropenia and was treated according to the local protocol with antibiotics, resulting in a rapid clinical recovery and negative cultures. The absolute CD34 + count was 18/µL on day 11 and 65/µL on day 12. On day 13, a large volume apheresis procedure was successfully performed, involving a minimum of 24 L for cell collection and an additional 2 L for plasma collection. This resulted in a yield of 15.4 × 10l^6^ CD34+/kg pre-cryopreservation and 9.4 × 10^6^ CD34+/kg post-cryopreservation, surpassing the minimum required amount of 6 × 10^6^ CD34+/kg post-cryopreservation [4]. The hCG + hCGβ levels normalized to < 2.6 E/L after the first chemo-mobilization cycle. Ten days after initiation of the second chemo-mobilization cycle, the patient developed pancytopenia (hemoglobin 6.8 g/dL, leucocytes < 100/µL, platelets 20 × 10^3^/µL). He received blood transfusions, resulting in improvement after approximately 3 days. Following the second chemo-mobilization cycle, LDH levels decreased to < 250 U/L. As a result, both hCG + hCGβ and LDH levels were normalized prior to HDCT.

Four weeks after initiating the second chemo-mobilization cycle, the patient received HDCT followed by ASCT rescue for three cycles, administered every four weeks. The conditioning regimen consisted of etoposide (400 mg/m2) and carboplatin (AUC 8), which were administered on three consecutive days, starting four days prior to the infusion of approximately 3.2 × 10^6^ CD34 + cells/kg. In addition, filgrastim was administered at a daily dose of 5 µg/kg starting from day + 1 after ASCT and continuing until the absolute neutrophil count reached or exceeded 1.0 × 10^3^/µL. HDCT was complicated by common adverse effects, including mucositis, bone marrow suppression requiring transfusions, and neutropenic fever. Neutrophil recovery was observed 10 days after ASCT in the first and second cycles, and 8 days after ASCT in the third cycle. After completion of treatment with the modified TC-CE regimen, his pre-existing grade 1 peripheral neuropathy progressed to grade 2, and he developed ototoxicity, necessitating the use of a hearing aid. The serum creatinine remained stable throughout treatment, indicating preservation of kidney function.

**Fig. 1:**
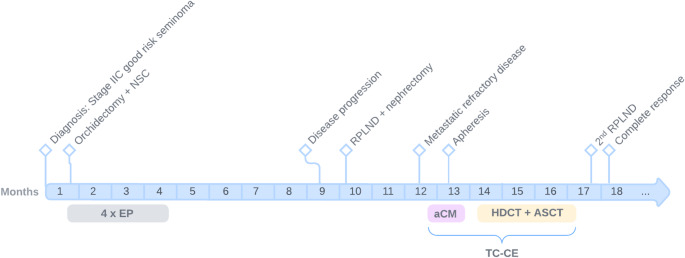
A timeline in months outlining the key events of this case report. NSC = nephrostomy catheter; RPLND = retroperitoneal lymph node dissection; EP = cisplatin and etoposide; TC-CE = modified TI-CE regimen; aCM = alternative chemo-mobilization using paclitaxel and cyclophosphamide; HDCT = high-dose chemotherapy using carboplatin and etoposide; ASCT = autologous stem cell transplantation

A PET-CT performed approximately six weeks after the third HDCT showed no metabolic activity, although retroperitoneal enlarged lymph nodes remained visible. Considering the patient’s history of rapidly recurring seminoma, a second RPLND was performed. This led to the successful resection of two lymph nodes exhibiting subtotal necrosis and the absence of malignant cells. Hence, the patient achieved a complete metabolic response, with no detectable tumor remnants post-RPLND. Currently, one year post-HDCT, there are no signs of disease recurrence. A timeline summarizing the main events of this case report is shown in Fig. 1.

## Discussion

Here, we present a case report of a patient with a refractory metastatic germ-cell tumor who underwent unilateral nephrectomy and was treated with the TC-CE regimen, derived from the TI-CE protocol. In this modified chemo-mobilization regimen, 2000 mg/m²/day ifosfamide was substituted by 1300 mg/m²/day cyclophosphamide, which did not compromise sufficient stem cell harvest. The first chemo-mobilization cycle already resulted in a significant stem cell harvest that exceeded the minimal required amount [3]. In addition, the tumor markers LDH and hCG + hCGβ were both normalized prior to initiation of HDCT. This indicates that the adapted chemo-mobilization regimen was effective in achieving both successful peripheral blood stem cell harvesting while preserving cancer treatment efficacy.

Within the TI-CE regimen, the nephrotoxic potential of ifosfamide is particularly notable [5, 6]. Important risk factors for the clinical manifestation of ifosfamide-induced nephrotoxicity in adults include high cumulative doses (>45 g/m^2^), unilateral nephrectomy, which might be attributed to a higher single-nephron load and previous exposure to cisplatin due to its additive nephrotoxicity [5, 6]. Ifosfamide was considered contraindicated in our patient, due to the presence of both unilateral nephrectomy and prior cisplatin exposure. Cyclophosphamide, a chemically related alkylating agent with a similar mechanism of action, exhibits much lower nephrotoxicity potential. This is attributed to the minimal biotransformation into the nephrotoxic metabolite chloroacetaldehyde and due to the lack of transport via the OCT2 pathway which prevents accumulation in proximal tubule cells [5, 9]. Cyclophosphamide demonstrates anti-tumor activity at standard doses in patients with germ-cell tumors, with no evidence of reaching a dose-response plateau [9–12]. Moreover, it effectively mobilizes hematopoietic stem cells into the peripheral blood, which is essential for successful stem cell harvesting [9, 13]. Thus, with appropriate dosing, cyclophosphamide can serve as an effective alternative to ifosfamide for chemo-mobilization in the TC-CE regimen after unilateral nephrectomy.

To our knowledge, cyclophosphamide has not been incorporated into chemo-mobilization regimens for patients with germ cell tumors. However, its use has been reported in consolidation treatment protocols [14–16]. Similarly, ifosfamide has been used in earlier studies as part of a tolerable conditioning regimen, administered at a dose of 10,000 mg/m² concomitantly with carboplatin and etoposide at dosing ranges of 900–2000 mg/m² and 1200–2400 mg/m², respectively [17, 18]. This regimen closely aligns with the conditioning regimen described by Lorch et al., consisting of 2200 mg/m² carboplatin, 1800 mg/m² etoposide and 6400 mg/m² cyclophosphamide [16]. It suggests that a dose of 10,000 mg/m² of ifosfamide can safely be substituted with 6400 mg/m² of cyclophosphamide. The TI-CE protocol later introduced a regimen that combines 6000 mg/m² of ifosfamide with 200 mg/m² of paclitaxel as chemo-mobilization regime [3]. Converting 60% of the 6400 mg/m² dose of cyclophosphamide results in an approximate cumulative dose of cyclophosphamide of 3850 mg/m². Alternatively, considering the metabolic pathways, 70–80% of cyclophosphamide is converted into active metabolites, whereas ifosfamide is converted into inactive metabolites for approximately 55% [5, 9]. Adjusting for this difference, a dose of 6000 mg/m² ifosfamide would theoretically be similar to 3900–4500 mg/m² of cyclophosphamide. Hence, we opted to replace 2000 mg/m²/day ifosfamide in the TI-CE chemo-mobilization regimen with 1300 mg/m²/day cyclophosphamide on three consecutive days, with the possibility to increase the dose in the second chemo-mobilization cycle if the initial harvest proved insufficient. However, dose adjustment was not required since CD34 + cell harvesting was already successful, eliminating the need for a second apheresis.

In the TI-CE regimen, aside from substituting ifosfamide with cyclophosphamide, the chemotherapeutic agents paclitaxel, carboplatin, and etoposide were administered without substitutions or dose adjustments. Paclitaxel, primarily metabolized by the liver and excreted through feces, is not strongly associated with nephrotoxicity, making dose modifications unnecessary [6, 19]. In contrast, carboplatin can accumulate in the proximal renal tubules, leading to potential nephrotoxicity. This risk is mitigated through hyperhydration and dose adjustments in patients with preexisting renal impairment [6]. The TI-CE regimen’s use of an AUC 8 model for carboplatin ensures dose adjustments based on current renal clearance [4]. Its primary toxicity, myelosuppression, is managed with ASCT post-HDCT [6]. Etoposide, which also predominantly causes myelosuppression and not nephrotoxicity, is mainly eliminated through renal excretion but did not necessitate dose adjustments due to sufficient renal clearance of the remaining kidney [6, 20]. Together, carboplatin and etoposide are the backbone of the HDCT regimen for germ cell tumors, and should therefore be maximally dosed for optimal treatment outcomes [4]. This approach resulted in a complete metabolic response, with no detectable tumor remnants. One year after HDCT, there are still no signs of disease recurrence.

## Conclusion

A modified, less nephrotoxic TC-CE regimen, in which ifosfamide was substituted with cyclophosphamide for chemo-mobilization, was successfully administered to a patient with refractory metastatic testicular seminoma post-unilateral nephrectomy. This approach enabled the collection of adequate stem cell yield in a single apheresis session and resulted in a complete metabolic response with no detectable tumor remnants, and no signs of disease recurrence one year post-HDCT. In conclusion, a dose of 6000 mg/m^2^ ifosfamide can be replaced by 3900 mg/m^2^ cyclophosphamide for chemo-mobilization.

## Data Availability

No datasets were generated or analysed during the current study.
